# Cyano-Phycocyanin: Mechanisms of Action on Human Skin and Future Perspectives in Medicine

**DOI:** 10.3390/plants11091249

**Published:** 2022-05-05

**Authors:** Daiva Dranseikienė, Gabrielė Balčiūnaitė-Murzienė, Jūratė Karosienė, Dmitrij Morudov, Nomeda Juodžiukynienė, Nataliia Hudz, Rima Jūratė Gerbutavičienė, Nijolė Savickienė

**Affiliations:** 1 Department of Pharmacognosy, Faculty of Pharmacy, Academy of Medicine, Lithuanian University of Health Sciences, Sukileliu av. 13, 50162 Kaunas, Lithuania; nijole.savickiene@lsmuni.lt; 2 Faculty of Pharmacy, Institute of Pharmaceutical Technologies, Academy of Medicine, Lithuanian University of Health Sciences, Sukileliu av. 13, 50162 Kaunas, Lithuania; gabriele.balciunaite@lsmuni.lt; 3 Laboratory of Algology and Microbial Ecology, Nature Research Centre, Akademijos St. 2, 08412 Vilnius, Lithuania; jurate.karosiene@gamtc.lt (J.K.); dmitrij.morudov@gamtc.lt (D.M.); 4 Department of Veterinary Pathobiology, Faculty of Veterinary, Academy of Veterinary, Lithuanian University of Health Sciences, Tilzes St. 18, 47181 Kaunas, Lithuania; nomeda.juodziukyniene@lsmuni.lt; 5 Department of Drug Technology and Biopharmaceutics, Danylo Halytsky Lviv National Medical University, Pekarska St, 69, 79000 Lviv, Ukraine; natali_gudz@ukr.net; 6 Department of Pharmacy and Ecological Chemistry, University of Opole, Kopernika pl. 11a, 45-040 Opole, Poland; 7 Department of Drug Technology and Social Pharmacy, Faculty of Pharmacy, Academy of Medicine, Lithuanian University of Health Sciences, Sukileliu av. 13, 50162 Kaunas, Lithuania; rima.gerbutaviciene@lsmuni.lt

**Keywords:** cyano-phycocyanin, cyanobacteria, skin diseases, wound healing, antimicrobial effect, antioxidative activity, anti-inflammatory effect, antimelanogenic effect, anticancer effect

## Abstract

Cyano-phycocyanin is one of the active pigments of the blue-green algae and is usually isolated from the filamentous cyanobacteria *Arthrospira platensis* Gomont (Spirulina). Due to its multiple physiological functions and non-toxicity, cyano-phycocyanin may be a potential substance for the topical treatment of various skin diseases. Considering that the conventional medicine faces drug resistance, insufficient efficacy and side effects, the plant origin compounds can act as an alternative option. Thus, the aim of this paper was to review the wound healing, antimicrobial, antioxidative, anti-inflammatory, antimelanogenic and anticancer properties and mechanisms of cyano-phycocyanin topical activities on human skin. Moreover, possible applications and biotechnological requirements for pharmaceutical forms of cyano-phycocyanin for the treatment of various skin diseases are discussed in this review.

## 1. Introduction

Over the past decade, researchers have focused their attention on microalgae and cyanobacteria [1]. Cyanobacteria, also known as blue-green algae, are the most primitive photosynthetic organisms on Earth which accumulate many active components with a broad range of important biological and pharmacological properties. Phycocyanins are light-absorbing biliproteins and biologically active compound that exist in all cyanobacterial species [2,3].

Phycocyanins are divided into three major types based on their spectral characteristics: cyano-phycocyanin (C-PC), derived from blue-green algae, R-phycocyanin (R-PC), derived from red algae, allophycocyanin, derived from both blue-green algae and red algae [4,5]. Wang L. and others (2014) also distinguished R-phycocyanin II (R-PC II) which is derived from marine cyanobacterium *Synechococcus* species [5]. 

Phycocyanins are oligomeric proteins composed of α and β subunits [4,5]. Each (αβ) monomer of C-PCs has three chromophores of phycocyanobilin. α Subunit carries one phycocyanobilin and the β subunit does two phycocyanobilins [5]. The molecular weights of α and β subunits are approximately 18 and 20 kDa, respectively. For R-PCs, an α subunit contains one phycocyanobilin but the β subunit carries one phycocyanobilin and one phycoerythrobilin [5]. Therefore, in the visual range of the light C-PCs, a one-peak absorption spectrum at approximately 610–620 nm is commonly shown, while R-PCs have two peaks in the absorption spectrum at wavelengths of 550 nm and 615–620 nm. The peak at approximately 550 nm comes from phycoerythrobilins and conspicuously expressed absorption maximum at approximately 615–620 nm is related to phycocyanobilins. Allophycocyanin has an absorption maximum at 650 nm [5,6,7,8]. R-PC from the marine red macroalga *Polysiphonia urceolata* (*Lightfoot ex Dillwyn*) *Greville* is composed of one α and two β subunits. Their molecular mass was 17.5 kDa, 21.3 kDa and 22.6 kDa, respectively. The α subunit had a strong absorption band from the chromophore of phycocyanobilin at a wavelength of the absorption maximum of approximately 597 nm and two β subunits which showed a strong absorption band from phycoerythrobilin at a wavelength of the absorption maximum of approximately 553 nm and an absorption shoulder from phycocyanobilin at a wavelength of approximately 600 nm [5].

C-PC is a hydrophilic and blue-colored biliprotein which is highly stable in the pH range of 5–8 [8]. Its isoelectric point ranges from 4.1 to 6.4. C-PC should be purified at a temperature of 4 ± 1 °C, as it is highly sensitive to heating [9]. 

A filamentous cyanobacteria *Arthrospira platensis* Gomont is the primary source of commercial C-PC [10]. In general, microalgae can grow in a broad range of habitats, pH levels and temperatures, and they have minimal growth requirements [11]. Kuddus M. and others distinguished four different options for C-PC production: photoautotrophic, mixotrophic, heterotrophic and recombinant production. They highlight that the cultivation under controlled conditions and low-cost downstream processing could be an economical advantage to meet the existing demand of C-PC [4]. The multistage process is required for C-PC isolation from algae and purification. First, variety of physical (sonication, cavitation, osmotic, repeated freezing and thawing) and chemical (usage of acids, alkali, detergents, enzymes) methods or their combinations are used for cell disruption. After cell breakage, the product is isolated from the supernatant by centrifugation. Purification is performed by extraction with ammonium sulfate, while separation is generally achieved by the column chromatographic methods using adsorbents, anion exchangers or gel filtration chromatography. Freeze drying is considered to be the most suitable method for pigment drying [12]. The purity of C-PC is usually established by dividing C-PC absorbance at a wavelength of its absorption maximum at 620 nm to a specific absorbance at a wavelength of 280 nm, which is related to the total protein, especially aromatic amino acids (A620/A280). The purity of 0.7 is assumed to be food grade, 3.9—as reactive grade and greater than 4.0—as analytical grade [4,9,13]. C-PC sparked attention due to its exceptional capability to show radical scavenging activity, slow down inflammatory processes, and reduce oxidative stress. Furthermore, it has been proven to have an anti-mutative, antimicrobial, antitumor, and wound healing properties [2,3,4,9].

**Figure 1:**
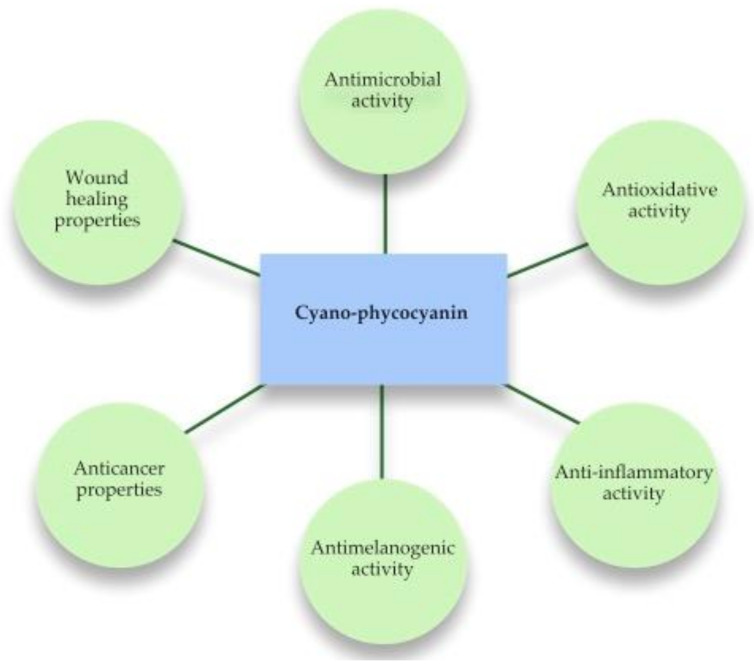
Physiological properties of C-PC for the topical application.

The aim of this paper was to review recent studies on the mechanisms of C-PC topical activities on human skin. Physiological properties of C-PC for the topical applications are given in Figure 1. Moreover, we discussed possible applications and biotechnological requirements for pharmaceutical forms of C-PC for the treatment of various skin diseases.

## 2. Biological Activity of C-PC

### 2.1. Wound Healing Effect

Skin acts as a barrier between the internal body and the external surrounding environment; therefore, wounds in the skin cause the loss of the integral anatomic continuity. Environmental conditions, physical accidents and general skin conditions, including but not limited to dryness and dermatitis, may contribute to skin wounds [1,14]. Cellular, molecular, biochemical, and physiological processes are a part of the skin recovery after the injury. The healing starts immediately following the arbitrary injury with the formation of a blood clot and the onset of inflammation. The proliferation and migration of dermal and epidermal cells, and a matrix synthesis occurs within 4 days, filling the wound area, and hence rebuilding the skin barrier. Finally, the tissue remodeling and maturing cycle enables a full recovery of the damaged skin tissue [15]. Some health conditions including but not limited to chronic diseases or bacterial infections could potentially undermine the recovery process [1]. Keeping this in mind, it is critical to identify bioeffective and natural ways of wound healing.

Madhyastha H.R. and others (2008) were the first to identify the effects of C-PC on wound healing. The effects of C-PC on fibroblast proliferation and urokinase-type plasminogen activator (uPA) migration were both investigated. It was demonstrated by in vitro studies that C-PC treatment (75 μg/mL) expedited the cell growth by inducing the G1 phase, thus resulting in an early event of cell cycle. It supports cell proliferation with no significant changes in the G2 or S phase. In addition, C-PC also increases the expression of cyclin-dependent kinases (cdK1 and cdK2), which play a significant role in the regulation of the cell cycle progression, ultimately leading to cell proliferation. This study determined that uPA is not considered as a significant molecule in controlling the proliferation and cell cycle in fibroblasts, but that it is involved in the cell migration process. The researchers also found that C-PC regulates uPA gene via cyclic adenosine monophosphate (cAMP) mediated mechanism that depends on protein kinase A (PKA) pathway [16]. 

The fibroblast cell motility in uPA condition induced by C-PC was greatly increased, however, it was ineffective in inducing cell motility in the absence of uPA. C-PC-induced fibroblast migration was greatly affected by uPA where migratory signals were facilitated by the Rho-family GTPases including Cdc 42 and Rac 1 via phosphoinositide-3 kinase (PI-3K) pathway. Cell migration and survival are greatly dependent on PI-3K which plays a key role in signal transduction leading to cellular processes [16,17]. The C-PC dose up to 100 μg/mL did not affect the viability of human fibroblast cells TIG 3-20, and the data confirmed that C-PC was not cytotoxic at doses sufficient to stimulate cell proliferation and migration. The wound healing effect of C-PC was also established by in vivo studies with mice [17]. The control and C-PC treatment groups did not show any significant differences in the initial phase of treatment, but it was observed that C-PC treatment achieved an 80% closure of the wound in contrast to 50% closure in the control models by the end of the first week [17].

Gur C.S. and others (2013) conducted in vitro and in vivo studies and confirmed the C-PC effect on wound healing. The effects of both Spirulina extract and C-PC isolated from crude Spirulina extract with a purity of 4.0 and higher were compared using cultured human keratinocyte and rat models. Proliferation, healing, and migration were increased in a dose-dependent manner. C-PC showed the best growth stimulation effect at 33.5 μg/mL dose. Additionally, in vivo study revealed that 1.25% of C-PC had a great impact on efficiency on the 7th day [18]. 

Therefore, considering increasing fibroblast proliferation and cellular migration towards the wound under the influence of C-PC, we can regard it as the active component of medicinal products for the treatment of external and internal wounds, for example, ulcers.

### 2.2. Antimicrobial Effect

The maintenance of the skin microbiome is important for the successful treatment of wounds and restoring the normal function of the skin. Bacteria, fungi, viruses, and mites are the major components of skin microbiota [19]. Healthy skin hosts a diversified community of microorganisms that inhabit specific areas of the body. For example, *Propionibacterium* species mainly are in the sebaceous glands. *Corynebacterium* and *Staphylococcus* species prevail in moist microenvironments [20]. The fungi of the genus *Malassezia* are at the core body and arm areas, while such a diverse combination of *Malassezia* spp., *Aspergillus* spp., *Cryptococcus* spp., *Rhodotorula* spp., *Epicoccum* spp., *Cryptococcus* spp., *Epicoccum* spp. and others colonized foot sizes [21]. In addition, acidic skin pH is determined by natural moisturizing factors and metabolites from skin commensal microbiota [19]. The skin microbiome acts as the physical barrier for the prevention of the invasive pathogens [21]. 

Various skin diseases including but not limited to those caused by bacteria, viruses or fungi may develop due to the skin microbiome and the immune system dysfunction. The imbalance between commensal and pathogenic microorganisms in the skin may even cause systemic diseases [21]. In such cases, it is important to select an appropriate antimicrobial therapy. The use of synthetic antimicrobials frequently faces the major problem of resistance. As a result, increased focus is on the antimicrobial agents of natural origin. 

#### 2.2.1. Antibacterial Effect

C-PC (16 μg/mL) from *Oscillatoria minima Gicklhorn* demonstrated the inhibitory effect against such bacteria as *Pseudomonas fragi*, *Escherichia coli*, *Pseudomonas vulgaris*, *Bacillus subtilis*, *Klebsiella oxytoca*, and *Streptococcus pyogenes*, while *Enterobacter aerogenes* and *Staphylococcus aureus* were not inhibited by C-PC [9].

Osman A. and others (2015) performed an in vitro study which revealed C-PC antibacterial activity against Gram-positive *Bacillus cereus*, *Staphylococcus aureus* and Gram-negative *Escherichia coli* and *Klebsiella pneumonia* bacteria strains. The inhibitory effect of C-PC (10–100 μg/mL) was compared with sterilized distilled water as the negative control and benzyl penicillin, clindamycin, ofloxacin and doxycycline as the positive controls. A concentration-dependent C-PC antibacterial activity was observed against before the mentioned bacterial strains. The activity against Gram-positive bacteria was slightly higher compared to that against Gram-negative ones. The anabaena C-PC (C-PC extracted from *Anabaena oryzae Fritsch*) concentrations corresponding to 50% inhibition of the tested bacteria (IC50): *Bacillus cereus*, *Staphylococcus aureus*, *Escherichia coli* and *Klebsiella pneumonia* were 30.56–31.25 μg/mL, while IC50 of the Spirulina C-PC (C-PC extracted from *Spirulina platensis*) for these bacteria were 33.32–40.96 μg/mL compared with benzylpenicillin: 35.93–221.2 μg/mL. These results suggest that Anabaena C-PC activity on Gram-positive bacteria is similar to benzylpenicillin, while relatively superior to Spirulina C-PC. The study also revealed that Anabaena C-PC induced morphological changes such as irregular wrinkled outer surface, fragmentation, adhesion, and aggregation in bacteria cell walls and membranes, but it had no specific activity on the biosynthesis of bacterial proteins [3]. Numerous studies showed that more than 90% of atopic dermatitis patients had *Staphylococcus aureus* colonization. *S. aureus* contributes to atopic dermatitis in a couple of ways. *S. aureus* can activate protease receptors in the disruption of the epidermal barrier by releasing endotoxins and enterotoxins. It is followed by mast cell stimulation, inflammation and keratinocyte dysregulation. Moreover, *S. aureus* upregulates the production of type 2 cytokines, such as thymic stromal lymphopoietin (TSLP), interleukin (IL)-4 and IL-13. High concentrations of IL-4 and IL-13 reduce keratinocyte-generated antimicrobial peptides. which are necessary to manage pathogenic organisms [20]. Therefore, the C-PC antibacterial action against *S. aureus* could be applied in the treatment of atopic dermatitis.

The study by Nihal B. et al. (2018) investigated the antimicrobial activity of C-PC extracted from Spirulina against the two major Gram-positive bacteria species linked to acne vulgaris: *Propionibacterium acne* (*P. acne*) and *Staphylococcus epidermidis* (*S. epidermidis*). *P. acne* is an anaerobic, rod-shaped bacteria present in the base of the hair follicle that breaks downs sebum and feeds on it [22]. The increase in these bacteria can lead to inflammatory processes in the skin. *P. acne* hyperproliferation in the sebaceous lipid environment generates reactive oxygen species (ROS) and proinflammatory compounds. The cytokine cascade injects the follicular wall structural changes of sebaceous glands and, therefore, causes variations in the sebum composition [1]. *S. epidermidis* is a part of normal skin microbiota and less founded in the mucosal flora. *S. epidermidis* might be found inside the acne vulgaris-affected pores. In the current study, two different formulations with C-PC were prepared, using water soluble and oleaginous bases; both were tested against the aforementioned bacteria. The formulation comprising a water-soluble base (PEG400, PEG4000, stearyl alcohol, glycerin, water) was superior to that one with the oleaginous base (paraffin hard, wool fat, cetostearyl alcohol, white soft paraffin, liquid paraffin). The study highlighted that, on the one hand, the water soluble base formulation had MIC values of 1.5 mg/mL and 1.8 mg/mL with a zone inhibition of 26.1 mm and 24.6 mm against *P. acne* and *S. epidermidis*, respectively. On the other hand, the oleaginous base formulation showed the MIC values as 1.6 mg/mL and 2.1 mg/mL, respectively, and the diameter of the bacteria growth inhibition as 23.4 mm and 21.3 mm, respectively [22]. These results might be caused by better C-PC solubility in water and, respectively, by better releasing C-PC from the dosage form. This also suggests that C-PC can be used as the natural component in hydrophilic ointments and creams for the treatment of acne.

Consequently, C-PC induces morphological changes in bacterial cell walls and membranes and is more effective against Gram-positive bacteria. It is worth noting that the purity of C-PC has a significant effect on antimicrobial activity.

#### 2.2.2. Antifungal Effect

An antifungal activity of the different concentrations and purity of C-PC from *Spirulina platensis* was compared by Muragan T. and others (2011). The purity of the crude extract was 1.17 and the concentration of C-PC was 0.8 mg/mL, while partially purified C-PC purity and concentration were 2.68 and 0.9 mg/mL, respectively. After purification by the micro-scale flash column chromatography, purified C-PC purity was 3.74 and concentration was 1.85 mg/mL. Two concentrations of 40 μg/mL and 80 μg/mL of the crude C-PC were tested for five fungal pathogens: *Candida albicans*, *Aspergillus niger*, *Aspergillus flavus*, *Penicillium species* and *Rhizopus* species. Neither of the concentrations inhibited the growth of the tested fungi. Meanwhile, the partially purified and column purified C-PC inhibited the growth at the minimum concentration 45 μg/mL. It was demonstrated that the purification improves fungi growth inhibition [23]. 

### 2.3. Anti-Oxidative Effect

Solar radiation reaching Earth is one of the factors that can affect and alter the skin barrier [24]. Ultraviolet (UV) radiation has both positive and negative impacts on the skin or body itself. Phototherapy has a strong effect on the endocrine system, rheumatoid arthritis, multiple sclerosis, inflammatory bowel diseases, body metabolism and psychological conditions. Moreover, phototherapy might also be used to treat various skin diseases [25]. It is important to note that prolonged direct sunlight and UV rays’ exposure, especially UVB radiation with the wavelength of 280–320 nm, can cause significant changes in the skin by promoting the production of ROS, including single oxygen, superoxide anion, hydrogen peroxide, hydroxyl, alkoxyl, and hydroperoxyl radicals. Under normal physiological conditions, ROS are generated during cellular respiration. The skin has an antioxidant defense system, which consist of antioxidant molecules such as vitamin C, E, glutathione, lipoic acid, uric acid, carotenes, coenzyme Q, etc. and enzymes. Enzymes with antioxidant activities include superoxide dismutase, catalase, peroxiredoxins, sulfiredoxins, thioredoxin and glutathione systems. One of their numerous functions is to prevent oxidative damages and cell death [26,27,28]. However, the abnormal ROS level increase caused by continuous UV exposure damages the body‘s antioxidant defense system, induces oxidative stress, and results in an injury of DNA, lipids, proteins and other cellular components [27,28,29]. These effects can influence different cellular processes in skin related to carcinogenesis and cancer progression, for example, cutaneous melanoma, or at least skin aging [26].

Numerous publications show that UV light is a significant factor both in malignant melanoma (MM) and non-melanoma skin cancer (NMSC) development. In vitro studies demonstrated that oxidative stress is elevated in melanoma cells [24,26]. Although classical theories agree that ROS provokes the initiation and progression of cancer, the role of ROS is ambiguous. Abundant evidence shows that ROS can act as tumor-promoting and tumor-suppressing agents [26]. Oxidative stress induced by disseminated melanoma cells in the circulating system potentially impedes the metastatic process. In addition, the antioxidant application greatly depends on the dose, dose-dependent redox balance and rebalance processes. Some antioxidants contribute to carcinogenesis prevention or, conversely, promote carcinogenesis. For example, some polyphenols such as rosmarinic acid or salicylic acid may have anticancer properties or cancerogenic effects depending on their concentration [26].

The antioxidant activity of C-PC is mostly measured by utilizing 2,2-diphenyl-1-picrylhydrazyl (DPPH) radical scavenging assay and 2,2′-azino-bis(3-ethyl benzothiazoline-6-sulfonic acid) diammonium salt (ABTS) radical scavenging assay. DPPH radical scavenging assay is related to the reduction in DPPH radical (deep violet colored) to yellow colored diphenyl picrylhydrazine in the existence of a hydrogen donor. Meanwhile, ABTS+ radical decolorization assay operates on the principle of producing ABTS^+^ by the oxidation of ABTS with potassium persulfate and reducing the occurrences of hydrogen-donating antioxidants [9]. Venugopal V.C. and coauthors (2020) evaluated the antioxidant activity of C-PC isolated from *Oscillatoria minima* and compared it with the butylated hydroxyanisole (BHA) antioxidant properties. The 1 mg/mL C-PC concentration showed 44% of DPPH radical scavenging activity, while BHA- showed approximately 90%. The same concentration of purified C-PC showed 95% of ABTS+ radical scavenging activity, while BHA was only approximately 70%. It might be presumed that the purified C-PC can decrease the formation of polar radicals and therefore, protect against different oxidative damages caused by ROS [9]. Another study performed by Gantar M. and coauthors (2012) evaluated the antioxidant activity of the purified C-PC isolated from the *Limnothrix* sp. strain 37-2-1 by DPPH method. The dry biomass of this strain contains 18% of C-PC, while, based on the literature data, the dry biomass of Spirulina constitutes 10–15% of C-PC. The researchers used the simple method for obtaining C-PC without the use of ion exchange chromatography. The procedure included the pigment precipitation from the cell lysate with an appropriate concentration of ammonium sulfate, purification with activated carbon and chitosan, and the sample concentration using tangential flow filtration. This method gave C-PC with a purity ratio of 4.3, which met the requirement for purity of analytical grade. The results revealed 100% antioxidant activity at the concentration of 0.15 mg/mL. These results also showed that the purity of C-PC increased the antioxidant properties. Moreover, the study demonstrated that cyanobacteria other than Spirulina can be a potentially superior source of C-PC [30].

Jang Y.A. and others (2021) tested the inhibition of ROS production by C-PC in UVB-induced human dermal keratinocyte cell line (HaCat cells). The UVB-induced ROS production was reduced by 51.2 and 55.1%, in cells treated with 40 and 80 μg/mL C-PC, respectively. Moreover, the same study revealed that C-PC (20, 40, 80 μg/mL) significantly reduced the matrix metalloproteinase-1 (MMP-1) and matrix metalloproteinase-2 (MMP-2) levels, which were elevated following the UVB radiation and associated with collagen degradation and photoaging. The increased expression of proteins involucrin, filaggrin and loricrin, which play an essential role in the formation of the skin barrier and hydration, was also observed after C-PC treatment [27]. These studies can be used for the development of creams for the treatment of the skin damages caused by sun light.

The in vitro study with human dermal fibroblasts and human epidermal keratinocytes showed that C-PC (1.0–20.0 μg/mL) can provoke the expression of heme oxygenase-1 (HO-1) at the mRNA and protein levels in a dose-dependent manner. HO-1 is an important molecule with inflammatory properties which is the host defense against oxidative stress. This activity of C-PC is mediated by the activation of nuclear factor erythroid-derived 2 (NF-E2) like 2 (Nrf-2) via phosphorylation of protein kinase C (PKC) α/β II. In addition, the expression of anti-apoptotic factor B-cell lymphoma 2 (Bcl-2) was significantly increased in UVB-irradiated cells after C-PC treatment, while the expression of the proapoptotic factors such as p53, Bax and caspase-3 was decreased. Moreover, the significant decrease in the DNA fragmentation in the UVB-irradiated cells was also observed in C-PC-treated cells [31].

These studies suggest that C-PC could be a potential antioxidant to slow down skin aging, including the formation of wrinkles, and even a promising tool in the treatment of skin cancer.

### 2.4. Anti-Inflammatory Effect 

Oxidative stress is mostly connected to the expression of cyclooxygenase-2 (COX-2), inflammation and lipid peroxidation in the cell membrane [26]. COX-2 catalyzes the conversation of arachidonic acid to prostaglandins and other eicosanoids. The overexpression of COX-2 is related to high levels of prostaglandin E2 (PGE2), which stimulates the proliferation of cells and mediates immunosuppression. Moreover, PGE2 plays a role in the inhibition of apoptosis by the upregulation of Bcl-2 gene; alternatively, arachidonic acid stimulates apoptosis. High levels of PGE2 are remarked in several malignancies including those of the bladder, pancreas, colon, cervix, breast, prostate, lung and skin. Meanwhile, C-PC inhibited COX-2 in a rat histiocytic tumor line AK-5 cells. Moreover, the same study revealed C-PC abilities to generate ROS in the tumor cells, activate caspase-3 and downregulate the expression of antiapoptotic substance Bcl-2, thereby making cancer cells vulnerable to apoptotic death. It is important to emphasize the fact that C-PC continued to induce apoptosis even after 24 h [32]. The main effects of COX-2 in cases of melanoma were also related to the augmentation of PGE2 production [33]. Some evidence has suggested that the overexpression of COX-2 is associated with tumor protein p53 mutation. Enache A.O. and coauthors (2018) observed the positive linear relation between p53 and COX-2 in a large proportion of basal cell carcinoma (BCC) cases [34]. Another study by Karahan N. and colleagues (2011) also confirmed that COX-2 expression is increased in BCC and suggested that COX-2 inhibition may be effective in BCC treatment, particularly for tumors with a higher level of COX-2 or a more aggressive phenotype [35]. Therefore, the use of several non-steroidal anti-inflammatory active substances with antiproliferative activity and selective COX-2 inhibitors such as celecoxib and C-PC could be a logical approach to prevent or treat cancer based on various studies [33].

The studies also showed that C-PC had stronger selective COX-2 inhibitory properties than celecoxib or rofecoxib. C-PC showed a positive effect in reducing levels of PGE2, and therefore, stimulated the proliferation of T and B immune cells and the immune response [36]. Other studies also confirmed that COX-2 plays a crucial role in the regulation of cell growth. The inhibition of COX-2 expression more significantly affected the growth suppression than inhibition of COX-2 catalytic activity. Consequently, these findings indicated the existence of two different COX-2 signaling pathways in cell growth regulation [37].

Gupta and others (2012) revealed that 50, 200 or 400 μg C-PC topical treatment effectively reduced 12-*O*-tetradecanoyl-phorbol-13-acetate (TPA) altered COX-2 and IL-6 expression in a dose-dependent manner. COX-2 is involved in the regulation of the IL-6 gene expression. The elevated levels of IL-6 increase the phosphorylation of the signal transducer and activator of transcription-3 (STAT3) protein. In addition, Il-6 plays a role in a tumor development and in several cellular processes [38].

Therefore, C-PC could be the natural anti-inflammatory agent with a stronger selective COX-2 inhibitory properties compared to celecoxib and rofecoxib. The inhibition of COX-2 and IL-6 expression, as well as the observed downregulation of Bcl-2 expression suggests that C-PC should be considered an acceptable anti-inflammatory and anticancer compound.

### 2.5. Antimelanogenic Effect

Skin pigmentation is an important defense mechanism against oxidative stress or UV radiation and is associated with the skin ageing. Abnormal hyperpigmentation in human skin may cause aesthetic problems and lead to serious skin diseases. The biosynthesis of melanin occurs in the specialized cytoplasmic organelles of melanocytes called melanosomes [39]. Melanogenesis is a multistage process that incorporates a number of enzymatic and chemical reactions occurring in the melanosomes [40]. Melanosomes contain the key enzymes which regulate the production of pigments such as tyrosinase (TYR), tyrosinase-related protein-1 (TYRP-1) and tyrosinase related protein-2 (TYRP-2). The upregulation of the expression of these key genes could promote melanogenesis in melanocytes and might be caused by the activation of the microphthalmia-associated transcription factor (MIFT) [41]. Three principal signal pathways for the regulation of melanogenesis can be distinguished: melanocortin-1 receptor (MC1-R) signaling pathway, the Wnt/β -catenin signaling pathway, the tyrosine kinase receptor KIT/stem cell factor (SCF) pathway. All these pathways activate the master regulator-MITF [40]. The cAMP/PKA signaling pathway is considered as the most significant signaling pathway of melanogenesis. The function of the melanocytes is modulated by the receptor melanocortin-1 (MC1-R). The binding of α-melanocyte-stimulating hormone (α-MSH) to MC1-R on the membrane of melanocytes activates adenylate cyclase and increases intracellular cAMP. In addition, this activates the PKA-cAMP response element-binding protein (CREB) pathway and then increases MITF and promotes melanogenesis. MC1-R plays a principal role in the regulatory process of skin pigmentation. Moreover, it is a melanoma susceptibility gene [41]. 

The studies revealed that many pharmacologically active substances can inhibit or stimulate melanin biosynthesis [39]. Wu L. and others (2011) studied the C-PC effect on α-MSH-stimulated melanogenesis and revealed that C-PC inhibited the tyrosinase activity and melanin formation depending on the dose (0.05 to 0.1 mg/mL). In addition, C-PC (0.1 mg/mL) significantly increased the accumulation of cAMP from 4.8 to 7.9 pmol/mL during the first 10 min. The researchers concluded that C-PC exerts cAMP-associated signaling to regulate melanogenesis via manipulating α-MSH-induced melanogenesis. The decrease in tyrosinase gene expression and melanin synthesis suggests the linkage between cAMP and mitogen-activated protein kinase (MAPK)/extracellular signal-regulated kinases (ERK) pathway. The C-PC-induced response of MAPK-ERK pathway-associated factors, ERK1/2 and MEK, were evaluated and it was determined that C-PC treatment significantly increased the phosphorylated ERK1/ERK 2 (p-ERK1/2) level and the phosphorylation of MEK after 540 min. Moreover, the expression of the MITF protein was also significantly inhibited at 540 min following C-PC treatment. It is known that ERK is associated with the degradation of MIFT. Moreover, C-PC inhibited the phosphorylation of p38 leading to the decline in phosphorylated-CREB. The hindrances of CREB phosphorylation as the transcription factor of MIFT leads to a subsequent reduction in MIFT transcription. Therefore, Wu’s and others’ (2011) study demonstrated that C-PC promoted the degradation of MITF protein through the upregulation of the MAPK/ERK signaling pathway, and the suppressed activation of CREB via the down-regulation of the p38 MAPK pathway [42]. 

The findings lead to consider C-PC as a potential melanogenesis inhibitor that can reduce skin pigmentation and aging processes caused by UV radiation. 

### 2.6. Anticancer Effect

Cancer is characterized by the uncontrolled growth and spread of abnormal cells. The main forms of skin cancer include basal cell carcinoma (BCC), cutaneous squamous cell carcinoma (cSCC) and melanoma. Melanoma is the most aggressive form of skin carcinomas, representing the principal cause of death associated with skin tumors [29]. Nowadays, the increasing number of melanoma skin cancer and MM cases is observed and mainly associated with an exposure to direct sunlight [24]. 

C-PC has more than one specific target in a cancer treatment and its antitumor effects might be demonstrated by the following mechanisms:

- Cell cycle suppression in the specific phases;
- The modification of the cellular redox state;
- The initiation of apoptosis and necrosis [32,43].

The apoptotic effect of phycocyanin was noticed in many in vitro and in vivo studies. Chromatin margination and condensation into dense granules or blocks, increasing percentage of cells in sub-G0/G1 phase, microvilli loss, membrane blebbing and cell shrinkage are the most commonly observed apoptotic features [44]. Studies have also confirmed that C-PC-induced apoptosis is mediated by the release of cytochrome C from mitochondria in the mouse macrophage cell line RAW 264.7. The treatment with 20 μM C-PC provoked the typical nuclear condensation and 16.6% of cells in the sub-G0/G1 phase. DNA fragmentation was observed in a dose-dependent manner. Moreover, the effect of C-PC on RAW 264.7 cells appeared to be associated with the reduction in PGE2 levels as the outcome of COX-2 inhibition [45]. C-PC antiproliferative action was justified by the accumulation of sub-G1 cell populations, DNA fragmentation and nuclear condensation in human melanoma A375 cells [46]. The study by Hao and others (2018) also showed a significant inhibition of the melanoma A375 cells growth after C-PC (6 μM) treatment, while the proliferation of HaCat cells was not influenced. C-PC antineoplastic effect on melanoma cells is associated with the downregulation of growth factor receptor-bound protein 2 (GRB2)-ERK1/2 pathway [47].

C-PC can promote the expression of Fas, a cell surface receptor, that directly triggers cell death by apoptosis [48], and intercellular cell-adhesion molecule-1 (ICAM-1) protein, while suppressing the Bcl-2 protein expression. Thus, the study revealed that C-PC could stimulate the activation of the pro-apoptotic gene and down regulate the expression of an anti-apoptotic gene. Furthermore, the study with HeLa cells noted the activation of caspases 2, 3, 4, 6, 8, 9 and 10 after C-PC treatment and suggested that C-PC-induced apoptosis was caspase-dependent [44]. The downregulation of Bcl-2 after C-PC treatment was also confirmed by another in vitro study with AK-5 tumor cells. This effect of C-PC may be significant because most the tumors are resistant to apoptosis because of the expression of Bcl-2. Thus, the downregulation of Bcl-2 may make the tumor cell more sensitive to other antineoplastic drugs. [32].

Recent in vivo investigation with mice tested the efficacy of C-PC in the representative genes of tumor development in mice skin. The tumorigenesis was induced by 12-0-tetradecanyol-phorbol-13-acetate (TPA) as a tumor-promoting agent. TPA induced the rate-limiting polyamine-biosynthetic enzyme ornithine decarboxylase (ODC). The elevated levels of ODC can play a role in epidermal tumorigenesis and are involved in cell cycle progression [38]. ODC can be induced by UVB and hormones and is greatly increased in SCC and BCC cases compared to levels in the normal skin tissue [49]. Topical applications with C-PC (50, 200 and 400 μg) showed an inhibitory effect (17, 38 and 50%, respectively) of TPA-mediated upregulation of ODC at the protein level and inhibitory effect of ODC (28.73, 42.89 and 43.06%, respectively) at the mRNA level [38].

Transglutaminase 2 (TG2) was another examined enzyme. This nuclear enzyme is dependent on Ca^2+^. TG2 catalyzes the transfer of primary amines into protein substrates. This enzyme induces apoptosis and cell differentiation. It can also inhibit the process of metastasis. The topical treatment with the same C-PC doses increased TG-2 protein level by 35.52, 141.2 and 219% and TG-2 mRNA 16.05, 50.67 and 59.60%, respectively [38]. It is quite well established that the overexpression of TG2 promotes the spontaneous apoptosis of cells or makes cells highly sensitive to apoptosis-inducing agents. However, there have also been studies indicating that an increased expression of TG2 may prolong cell survival by preventing apoptosis as TG2 causes the constitutive activation of NF-KB in cancer cells and TG2 could be an attractive alternate target for inhibiting constitutive NF-KB activation and rendering cancer cells sensitive to anticancer drugs [50]. Therefore, studies regarding the connection of TG2 and apoptosis are controversial. Some authors highlighted that TG2 dual function, antiapoptotic or a proapoptotic ones, depends on the cell type and its location within the cell. The high concentrations (>1 mM) of Ca^2+^ induces the activation of TG2, which promotes the inter- and intramolecular cross-linking of proteins and causes cell death, while low levels (<1 mM) of Ca^2+^ and high concentrations (>9 μM) of guanosine triphosphate (the conditions that generally predominate inside cells) promotes the signaling of cell-survival mediated by TG2 [51]. The isopeptide bond reactions catalyzed by transglutaminases have great physiologic significance in the human body. Isopeptide bonds are frequently found in hair and skin. They accumulate during wound healing, apoptosis, and blood clotting. Several pathologic conditions, such as neurodegeneration, progressive tissue fibrosis, autoimmune diseases, infectious diseases, and ailments related to the integrity of the stratum corneum of the epidermis can be provoked by the dysregulation of transglutaminase functions [52]. TG2 expression proved to be positive in regulating the number of cancer cell types, especially in tumors with high resistance to chemotherapeutic drugs or metastasis [51]. The biologically active substances, that can affect tumor-encoded genes whose expression contributes to the development of drug resistance and metastasis may be significant in the cancer treatment. Chhabra A. and others (2009) also accentuated that the therapeutic efficacy of anticancer drugs can be increased by the downregulation or inhibition of TG2 by weak interfering RNA or by small molecule inhibitors [51].

Gupta N.K. and others studied the C-PC effect on the TPA-altered expression of phosphorylated signal transducer and activator of transcription-3 (pSTAT3) and confirmed the antitumor properties of C-PC. TPA application increased the level of pSTAT3 compared to the control group, while in the presence of C-PC, this TPA-caused upregulation of pSTAT3 protein was inhibited depending on the dose. It was established that IL-6 promotes the activation of STAT3, and accordingly, tumor development [38].

In summary, it can be stated that C-PC has clear potential in cancer treatment due to its antioxidative properties, the ability to inhibit COX-2 expression, reduce PGE2 level and induce apoptosis in tumor cells. Considering that C-PC can activate the pro-apoptotic gene and downregulate the expression of the anti-apoptotic gene, C-PC could be combined with other anticancer drugs, reducing the effective dose of anticancer drugs, minimizing side effects and tumor resistance to chemotherapeutic treatment.

### 2.7. Summary of C-PC Topical Activities

The discussed investigations of C-PC topical activities are given in Table 1.

## 3. Advanced Technology for Functionalization of C-PC

As a barrier between the internal body and the external environment, the skin may be affected by many conditions such as viral, bacterial, fungal and parasitic infections or UV radiation, that can result in various disorders such as trauma, inflammation, rashes, pigmentation disorders, tumors and even cancer [53]. Nowadays, a wide range of therapeutic methods are used for skin disorders; nevertheless, some of them are limited by adverse reactions, skin barrier or resistance [54]. 

For example, the excessive use of cleansing cosmetics can disturb the hydrophilic barrier and natural microbiota, cause skin irritations and lead to the atopic skin conditions [54]. This situation is often observed in the inappropriate treatment of acne or self-medication. Acne is a long-term skin condition that occurs when hair sacs become clogged with dead skin cells. It is categorized into non-inflammatory (characterized by spots, blackheads, whiteheads) and inflammatory (characterized by red and swollen pimples) types [22]. Acne can also be treated with prescription creams and gels that inhibit pore swelling and reduce external inflammation. In addition, isotretinoin may be prescribed in some cases. However, treatment with isotretinoin is strictly contraindicated in pregnant and lactating women because of its teratogenic effect. For these reasons, there is a growing interest in the natural effective remedies that can have complex impact on acne skin. 

Antioxidant, antimicrobial and anti-inflammatory effects of C-PC suggest its use in acne treatment. Nival B. and others (2018) tested two different formulations with C-PC as topical anti-acne preparations. The oleaginous base consisted of hard paraffin, wool fat, cetosstearyl alcohol, soft paraffin, liquid paraffin and C-PC extract. Meanwhile, water-soluble base was composed of PEG400, PEG4000, stearyl alcohol, glycerin, water and C-PC extract. The consistency of the water-soluble base was superior to that of an oleaginous base because of better spreadability characteristics. Moreover, in vitro drug form diffusion of water-soluble base formulation was found to be at 92% after 5 h compared to oleaginous base formulation only at 88%. Water-soluble base formulation also demonstrated higher antibacterial activity against *P. acne* and *S. epidermidis* [22]. These results can be also explained by good C-PC solubility in water. Moreover, mineral oils such as paraffins and silicone oils can also promote fat accumulation in the skin, therefore, water-based formulations may be more appropriate for acne treatment.

The skin is highly effective selective penetration barrier. The multilayered membrane allows the substances to penetrate via passive diffusion to some extent. However, only relatively low molecular weight (<500 Da) substances with an adequate lipophilicity can penetrate via passive diffusion into the deeper skin layers or through the skin. Meanwhile, peptides and proteins are poorly absorbed because their molecules are generally large and hydrophilic [55].

Successful topical treatment depends on the active substances. Moreover, the inactive ingredients through which the drug is delivered to the skin and the type of application modality play a crucial role. In clinical practice, there are many limitations associated with semi-solid formulations, such as ointments, creams, oils or gels. Localized side effects and insufficient skin penetration often become a major challenge in development of new forms for topical use. For these reasons, nanotechnology can be an efficient system for cutaneous delivery in the treatment of skin diseases and in cosmetics [53].

The use of natural compounds and their transport in pharmaceutical forms are capable of controlling the release and absorption can be used as an effective treatment way for various skin diseases. For example, lipid-based nano-systems have a structural similarity to lipids in the skin. This similarity promotes the interaction between the lipid matrix of the nano-system and the epidermis intracellular lipids. It improves the skin hydration and penetration of the encapsulated molecules [53].

It was observed that liposomes can become a great carrier for the topical delivery of large molecular weight proteins such as C-PC. The use of traditional phospholipid vesicles improved the in vivo anti-inflammatory activity depending on the dose. Moreover, the use of liposomes allows to halve the C-PC dose in order to produce the same anti-inflammatory effect as free protein [55]. However, the penetration enhancer-containing vesicles (PEVs), especially propylene glycol, improved the dermal and transdermal delivery of the high molecular weight protein C-PC compared to the conventional liposomes and ethosomes [56]. Castangia I. and others (2016) study revealed that phycocyanin encapsulated into hyalurosomes, especially PEG-hyalurosomes, can penetrate the deeper skin layers and show better antioxidative properties on stressed human keratinocytes [57]. New biocarriers, named santosomes, were also suggested by Castangia I. and others (2016). Nanovesicles are formed of hydrogenated phosphatidylcholine, cholesterol and the essential oil of *Santolina insularis (Gennari ex Fiori) Arrigoni*. Propylene glycol was added as a hydrophilic penetration enhancer, and C-PC was incorporated into the modified nanovesicles. *S. insularis* belongs to *Asteraceae* family and is an endemic plant of Sardinia. The plant accumulates mono- and sesquiterpenes, which are good penetration enhancers able to improve the absorption of different substances through the skin. After toxicity assessment, the optimal dilution was selected: 100 μg/mL of phosphatidylcholine, 50 μg/mL of C-PC and 25 μg/mL of essential oil. C-PC was capable of entering human keratinocytes when delivered by santosomes even after 1 h of treatment and stayed consistently high for 24 h, while free C-PC was unable to enter the cells. In addition, the delivery by santosomes prolonged the antioxidative and anti-inflammatory effect of C-PC and its ability to restore, repair and protect the skin [58]. Skin restoration and wound healing are complex physiological processes consisting of multiple stages and reconstruction. Wound healing, especially burns, diabetic ulcers and bedsores, are a big challenge for a healthcare system. Dev A. and others (2020) suggested an injectable hydrogel from natural polysaccharide κ-carrageenan (κ-CRG) and C-PC. Antimicrobial, antioxidant, anti-inflammatory and wound healing effects of C-PC were accommodated for this injectable and regenerative wound dressing matrix. κ-CRG-C-Pc hydrogel improved fibroblast migration, blood clotting and wound closure capabilities. Furthermore, κ-CRG hydrogel was degraded within twenty-four hours after application, while κ-CRG-C-Pc hydrogel remains in the wound for six days. The best results of wound healing were observed after eight days of treatment with κ-CRG-C-Pc hydrogel. In addition, the κ-CRG-C-Pc hydrogel treatment group showed maximum collagen synthesis and superior hemostatic capabilities that could help in wound closure and tissue restructuring in the wound site [59]. Madhyastha and others’ (2020) in vitro study with the skin fibroblast cells revealed that C-PC conjugated with the silver nanoparticles (AgcPCNPs) increased the fibroblast migration towards the wound area. This nanoparticles form combined the wound healing properties of C-PC and the broad-spectrum antibacterial activity of the silver ions [60].

Polylactic acid (PLA) patches with C-PC were utilized for the treatment of less complicated wounds. PLA was chosen as a nontoxic biodegradable polymer which possesses similar properties to conventional plastic patches, while C-PC was used as an active ingredient with antioxidant and anti-inflammatory properties. Alginate was embedded in the PLA matrix with C-PC to create prolonged release patches (effective for approximately 20 h). The patches had the best properties (elasticity, release of C-PC) while the percentage ratio of C-PC and alginate components in the patch was 40/60 [61].

The engineered biosilica could be beneficial as a mesoporous biomaterial for anticancer drug delivery. Diatoms are unicellular eukaryotes, performing photosynthesis. Their cell walls are formed of amorphous SiO_2_ and called frustules. These silica frustules are characterized by specific pores as small as 20 nm and can be applied as nanocarriers. C-PC combination with mesoporus biosica improved C-PC stability and biocompatibility and was effective in photodynamic tumor cells therapy. Even the highest concentrations of free C-PC or functionalized biosilica caused below 10% of RAW264.7 cell death without illumination. Thus, the results demonstrated the safety of free C-PC and C-PC modified biosilica. Samples with 150 μg/mL C-PC and light irradiation had less than 25% of cell death, while under the same illumination, functionalized biosilica (5–150 μg/mL) showed 66–81% of RAW264.7 cell death, respectively. This study introduces a new field of C-PC biomedical applications [62]. Jiang L. and others (2019) also suggested a novel cervical tumor targeted nano-drug. Carboxymethylchitosan (CMC) served as a nanomaterial for the nano-formulation construction. CMC is well soluble in water, biodegradable, biocompatible and a non-toxic substance. Thus, the combination of C-PC and CMC improved the C-PC stability, ensured the slow release of C-PC and increased the C-PC efficacy in cancer treatment. Moreover, CD59 specific ligand peptide (CD59sp) was used to act as a targeted molecule which selectively combines CD59. It is known that CD59 is elevated in many solid tumors and lowly expressed in normal cells. CD59 takes part in suppressing the formation of a complement attack complex (MAC). As a result of this, suppressing tumor cells avoid the response of the immune system. Thus, C-PC/CMC-CD59sp nanoparticles (C-PC/CMC-CD59spNPs) significantly provoke G0/G1 cell cycle arrest in cervical cancer HeLa and SiHa cells compared to C-PC and C-PC/CMC nanoparticles. The cell proliferation was inhibited in a dose-dependent manner. Nanoparticles with C-PC induced early apoptosis in both HeLa and SiHa cells, increased the expression of cleaved caspase-3 protein, and decreased the expression of bcl-2 protein; however, C-PC/CMC-CD59spNPs inhibited MMP-2 protein expression and the growth of cervical cancer tumor more significantly [63].

Due to its complex effects, C-PC is increasingly being considered as a potential biological active substance in skin diseases, even including cancer. As other proteins, C-PC requires more attention during manufacture/formulation and the administration processes because of its unique structure and unstable physical-chemical properties [19]. According to most authors, pH and temperature are the key factors affecting the stability of C-PC [11]. It can be noted that the optimum pH range for C-PC preparations is 5.5–6.0 [64]. At values close to temperatures of 25–45 °C, the C-PC solution degrades very slowly. The degradation of C-PC is initiated at temperature above 45 °C, and the denaturation of protein is accelerated at temperatures above 70 °C [65]. It is observed that the stability of C-PC decreases with time and temperature; however, the reactive grade C-PC showed significantly lower thermal stability than food-grade C-PC [65]. 

Moreover, C-PC is sensitive to light, therefore, the best storage conditions should be in the dark [65]. 

As C-PC is very sensitive to environmental stress, various additives are used to enhance the stability of C-PC. The chemical structure and the concentration of the preservatives must be safe for humans. Moreover, they must not denature protein or adversely affect its properties. Mono- and disaccharides (glucose, fructose, saccharose, trehalose, lactose, maltose and sorbitol), and organic acids (citric acid, ascorbic acid, and benzoic acid) or inorganic salts (sodium chloride, calcium chloride) may be used as preservatives [65]. Many studies focus on the additives that can increase the thermostability of C-PC [11]. The addition of glucose could enhance the activation energy up to 4 times due to the polymerization of protein C-PC by sugar, and prevent the damage of the C-PC structure by heating [66]. Moreover, 2.5% sodium chloride is also considered as an efficient preservative for C-PC stability [64]. The mentioned instability of C-PC limits its use in medical applications and the development of new products. Therefore, the processes with no heating, no ultrasound, and no organic solvents should be prioritized. Moreover, another way preventing the proteins degradation may be the usage of encapsulated forms (nanofibers, microparticles, or nanoparticles) [65]. The employment of aqueous two-phase systems using polymer/salt (4% PEG and 18% salt, w/w) and polymer/polymer (6% PEG and 10% dextran, w/w) as a carrier material for C-PC double encapsulation increased the stability and the purity of C-PC. Moreover, this double encapsulation extended the shelf life of C-PC for the 6 months without markedly changing the powder characteristics [67]. Guo J.W. and others (2021) also confirmed the effectiveness of chemical penetration enhancers, transferosomes, nanoparticles delivery system, or cell-penetrating peptide-modified nanocarriers in transdermal delivery of proteins. In addition, the same researchers warned that larger molecular weight peptides or proteins may cause the inflammation. Therefore, the appropriate dose of protein/peptides should be very responsibly chosen in the topical delivery system [19].

Thus, C-PC is hydrophilic protein highly sensitive to environmental conditions, especially pH and temperature. Maintaining pH between 5.5 and 6.0 and the temperature under 45 °C is required for its stability. Preservatives such as glucose, fructose and sodium chloride help prevent the degradation of protein. The use of modern nanotechnologies may be the key in topical applications of C-PC for the treatment of various skin diseases. 

## 4. Conclusions

Studies have revealed that C-PC significantly contributes to wound healing and tissue regeneration by inducing fibroblast proliferation and enhancing cellular migration. In addition, C-PC induces morphological changes in bacterial cell walls and membranes and is more effective against Gram-positive bacteria. Due to the inhibition of ROS production and antimelanogenic properties, C-PC can be considered a potential compound to slow down the aging process of skin. It is worth noting that the purity of C-PC has a significant effect on antimicrobial and antioxidative activity. C-PC is a natural anti-inflammatory agent with a stronger selective COX-2 inhibitory properties compared to celecoxib and rofecoxib. Based on the reviewed studies, it can be stated that C-PC has clear potential in cancer treatment due to its antioxidative effects, its ability to inhibit COX-2 expression, reduce PGE2 level and induce apoptosis in tumor cells. Considering that C-PC could increase the activation of pro-apoptotic genes and downregulate the expression of anti-apoptotic genes, C-PC could be further investigated for the treatment of skin cancer as monotherapy or in combination with other anticancer agents to reduce tumor resistance to chemotherapy treatment and its side effects. The adoption of modern nanotechnologies and stabilizers usage could ensure C-PC stability, the penetration of key skin layers and applications of C-PC to the topical treatment of various skin diseases.

## Figures and Tables

**Table 1:** Investigations of C-PC topical activities.

No	Study Profile	Origin of C-PC	Dose/Concentration of C-PC	Activity	Reference
1	WI-38 fibroblast cells	*Spirulina fusiformis Voronichin*	25, 50, 100 μg/mL	uPA gene regulation through cAMP-mediated PKA pathway	Madhyastha et al. (2006) [16]
2	Human fibroblast cells TIG 3-20	*Spirulina fusiformis*	10–200 μg/mL	wound healing promotion by upregulating uPA, GTPases Cdc 42 and Rac 1 stimulation through PI-3K pathway fibroblast proliferation induction via the cyclin-dependent kinases cdK1 and cdK2 cellular migration towards the wound enhancement in a uPA-dependent manner	Madhyastha et al. (2007) [17]
3	Male mice	*Spirulina fusiformis*	75 μg/mL	wound area reduction	Madhyastha et al. (2007) [17]
4	Human keratinocytes	*Spirulina platensis*	0.0335, 0.335, 3.35, 33.5 μg/mL	proliferation, healing, and migration promotion	Gur et al. (2012) [18]
5	Male rats	*Spirulina platensis*	1.25, 2.5%	wound healing process improvement	Gur et al. (2012) [18]
6	*Pseudomonas fragi* *Escherichia coli* *Pseudomonas vulgaris* *Bacillus subtilis* *Klebsiella oxytoca* *Streptococcus pyogenes* *Enterobacter aerogenes* *Staphylococcus aureus*	*Oscillatoria minima*	16 μg/mL	antibacterial activity against *Pseudomonas fragi*, *Escherichia coli*, *Pseudomonas vulgaris*, *Bacillus subtilis*, *Klebsiella oxytoca*, *Streptococcus pyogenes*	Venugopal et al. (2020) [9]
7	*Bacillus cereus* *Staphylococcus aureus* *Escherichia coli* *Klebsiella pneumonia*	*Anabaena oryzae*	10, 25, 50, 75, 100 μg/mL	antibacterial activity morphological changes in the cell walls and membranes	Osman et al. (2015) [3]
8	*Propionibacterium acne* *Staphylococcus epidermidis*	*Spirulina platensis*	10% C-PC extract of the formulation of oleaginous base compared with the same concentration of water-soluble base	antibacterial activity	Nihal et al. (2018) [22]
9	*Candida albicans* *Aspergillus niger* *Aspergillus flavus* *Penicillium species* *Rhizopus species*	*Spirulina platensis*	40–80 μg/mL	antifungal activity	Murugan et al. (2011) [23]
10	-	*Oscillatoria minima*	1 mg/mL	anti-oxidative activity (DPPH, ABTS methods)	Venugopal et al. (2020) [9]
11	-	*Lamotrix sp. 37-2-1* *Spirulina platensis*	0.05–0.3 mg/mL	anti-oxidative activity (DPPH)	Gantar et al. (2012) [30]
12	Human dermal keratinocyte (HaCat) cells	*Spirulina platensis*	5, 10, 20, 40, 80 μg/mL	ROS production inhibition protection against UVB-induced damage MMP-1 and MMP-9 expression inhibition involucrin, filaggrin and loricrin expression promotion	Jang et al. (2021) [27]
13	Human dermal fibroblasts Human epidermal keratinocytes	-	1–20 μg/mL	protection against UVB-induced apoptosis HO-1 expression induction Nrf-2 via phosphorylation of PKC α/β II activation	Kim et al. (2018) [31]
14	Rat histiocytic tumor cells AK-5	*Spirulina platensis*	30 μM	DNA fragmentation and apoptosis in tumor cells induction Bcl-2 down regulation	Pardhasaradhi et al. (2021) [32]
15	Female mice	*Spirulina platensis*	50, 200, 400 μg/mL	COX-2 and IL-6 expression reduction ODC, pSTAT3 downregulation TG2 upregulation	Gupta N.K. and Gupta K.P. (2021) [38]
16	Murine melanoma cells	*Spirulina platensis*	0.05, 0.1, 0.2 μg/mL	tyrosinase activity, melanin production, p38 phosphorylation, MIFT inhibition cAMP accumulation p-ERK1/2 level, phosphorylation of MEK induction	Wu et al. (2011) [42]
17	RAW 264.7 mouse macrophage cell line	*Spirulina platensis*	20 μM	apoptosis induction nuclear condensation DNA fragmentation accumulation of sub-G1 cell populations COX-2 inhibition	Reddy et al. (2003) [45]
18	A375 melanoma cells	-	6 μM	melanoma cells inhibition GRB2-ERK1/2 pathway downregulation	Hao et al. (2018) [47]
19	HeLa cells	*Spirulina platensis*	Different concentrations	apoptosis induction cells in sub-G0/G1 phase augmentation increased expression of Fas and ICAM-1 caspases 2, 3, 4, 8, 9, 10 activations Bcl-2 downregulation	Li et al. (2010) [44]

## Data Availability

The data are contained within the article.
